# Real-time observation of neutrophil extracellular trap formation in the inflamed mouse brain via two-photon intravital imaging

**DOI:** 10.1186/s42826-022-00126-3

**Published:** 2022-06-13

**Authors:** Da Jeong Byun, Young Min Kim, Young-Min Hyun

**Affiliations:** 1 Department of Anatomy, Yonsei University College of Medicine, Seoul, Republic of Korea; 2 BK21 PLUS Project for Medical Science, Yonsei University College of Medicine, Seoul, Republic of Korea; 3 Department of Medicine, Yonsei University College of Medicine, Seoul, Republic of Korea

**Keywords:** Two-photon microscopy, Intravital imaging, Brain, Neutrophil, Neutrophil extracellular trap

## Abstract

Intravital imaging via two-photon microscopy (TPM) is a useful tool for observing and delineating biological events at the cellular and molecular levels in live animals in a time-lapse manner. This imaging method provides spatiotemporal information with minimal phototoxicity while penetrating a considerable depth of intact organs in live animals. Although various organs can be visualized using intravital imaging, in the field of neuroscience, the brain is the main organ whose cell-to-cell interactions are imaged using this technique. Intravital imaging of brain disease in mouse models acts as an abundant source of novel findings for studying cerebral etiology. Neutrophil infiltration is a well-known hallmark of inflammation; in particular, the crucial impact of neutrophils on the inflamed brain has frequently been reported in literature. Neutrophil extracellular traps (NETs) have drawn attention as an intriguing feature over the last couple of decades, opening a new era of research on their underlying mechanisms and biological effects. However, the actual role of NETs in the body is still controversial and is in parallel with a poor understanding of NETs in vivo. Although several experimental methods have been used to determine NET generation in vitro, some research groups have applied intravital imaging to detect NET formation in the inflamed organs of live mice. In this review, we summarize the advantages of intravital imaging via TPM that can also be used to characterize NET formation, especially in inflamed brains triggered by systemic inflammation. To study the function and migratory pattern of neutrophils, which is critical in triggering the innate immune response in the brain, intravital imaging via TPM can provide new perspectives to understand inflammation and the resolution process.

## Background

Technological advancements in biology have led to the development of cutting-edge imaging tools that use high-resolution images and videos to present biological phenomena in immense detail. Intravital imaging via two-photon microscopy (TPM) is a prime example of such an advancement. Using this technology, researchers can now explicitly visualize biological events and overcome previous experimental limits in dealing with delicate or complex cells and organs, such as the brain. Furthermore, spatiotemporal and real-time observations of actual pathological events have become possible. In this review, we demonstrate how two-photon intravital imaging can be used in the field of immunology research, particularly in the brain. In addition, we outline research on neutrophil extracellular traps (NETs), a novel feature of neutrophils, and how intravital imaging plays an important role in understanding NET formation.

## Main text

### Two-photon microscopy

TPM is a powerful technique to visualize live dynamics. Compared with conventional single-photon microscopy, TPM exploits two nonlinear photons, eliminating several fundamental limits. Specifically, the photons used in TPM have a long wavelength of approximately 700–1,000 nm that can pass through deep tissue (approximately 1,000 μm) [1–3]. This enables lengthy imaging sessions while inflicting minimal tissue damage with less phototoxicity [4, 5]. By utilizing fluorescence-labeled antibodies or genetically modified fluorescent mice, this technique provides optical sectioning, which provides anatomical information of the target area. These areas include specific types of cells, the bloodstream, and specific structures in the bodies of live animals. Furthermore, second-harmonic generation (SHG) is a collateral phenomenon of TPM in which two photons with the same frequency interact, generating a new photon with twice the optical frequency of the original photons. This allows researchers to observe different types of spatially organized structures, such as collagen, cholesterol crystals, and bony structures without specific fluorescence markers. Indeed, physiological and etiological phenomena have been observed in real time by TPM [6]. Taking advantage of these features, intravital imaging using two-photon microscopy has been employed in numerous studies involving laboratory animals [4, 5, 7]. Live-cell movements, microparticles, and structures have been monitored by staining fluorescent antibodies, plasma markers, or genetically modified mice that express fluorescent proteins [8–10]. Thus, two-photon intravital imaging enables the elucidation of biological mechanisms in diseased organs of live animals (e.g., brain, lung, trachea, liver, kidney, spleen, dermal tissue, and cochlea) [11–16]. In summary, two-photon microscopy provides structural and functional information from a spatiotemporal viewpoint in various organs, without physical invasion, making it a revolutionary tool to observe biological events and suggest novel therapeutic approaches.

## Brain intravital imaging

The brain is one of the most delicate and enigmatic organs in humans. Therefore, the field of neuroscience has used animal models for the intense study of largely elusive biological processes in the brain. Histological observation of a separated brain is widely used in conventional studies, although it is confined to a fixed single time period. With this method, it is difficult to monitor the cerebral dynamics of an intact brain and speculate on physiological reactions in real time. However, applying TPM in this field ameliorates the previous experimental limitations with several benefits, as described in the previous section. This enables us to observe the spatiotemporal dynamics of targeted cells and inner structures in real-time by tagging specific fluorescent antibodies or modifying genes that indigenously contain fluorescent proteins [17–23]. Furthermore, even though the brain is known as an immune-privileged organ, it has been noted that leukocyte migration and infiltration are pivotal events in the neuroinflammatory state. As an example, a neutrophil is a well-known leukocyte that reacts during an early phase of inflammation by infiltrating the brain to resolve inflammation therein [22, 24]. However, some aspects remain ambiguous, such as the effects or functions of the remaining leukocytes. Accordingly, it is becoming increasingly important to observe biological dynamics in real time. Therefore, employing TPM can provide new perspectives in brain studies. Herein, we used a mouse brain model to illustrate the experimental setup and application of TPM intravital imaging with a detailed protocol [25].

**Fig. 1:**
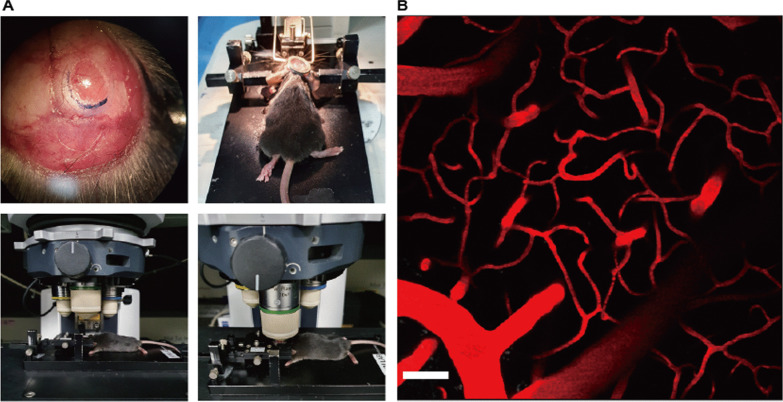
Brain intravital imaging. **A** Each image represents the setup of mouse brain imaging with the chamber for two-photon intravital imaging. Anesthetized mouse was put in a customized chamber to hold the skull and maintain body temperature. A craniotomy was conducted to expose the cortex surface. The exposed cortex was covered with cover glass (3–5 mm), and the metal ring was placed parallel to the brain structure (upper panels). The cranial window of the mouse brain was connected to a water-immersed lens for imaging capture. Next, the lens in the cranial window was immersed in PBS (bottom panels). **B** A snapshot of two-photon intravital imaging of the mouse brain is displayed. The bloodstream in this imaging was visualized with Texas Red-dextran (70 kDa, 2.5 mg/kg). Scale bar: 50 μm

It is critical to expose and prepare the target organ with minimal harm to employ TPM because damage can make it difficult to observe perturbations in intravital imaging. First, the mouse was prepared with an adequate anesthetic dosage (ketamine/xylazine or Zoletil) via intraperitoneal injection. Next, the mouse was placed in a custom-designed chamber to hold the skull and maintain body temperature while performing the operation. The skin of the head was removed from the lambda to the bregma region of the calvaria (Fig. 1A). The periosteum was peeled off, and the targeting position was marked on the parietal bone according to the size of the round cover glass. The mark was bored cautiously using a micro drill, washed, and cooled down frequently with PBS buffer. The exposed cortex surface was sealed with a round coverslip using tissue adhesive while remaining hydrated with PBS (Fig. 1A). A metal ring (i.e., an angel ring) was attached using dental cement around the cranial window to create a barrier for sustaining water during intravital imaging. Fluorescent markers, such as the dextran family and specific antibodies against any epitope of cells in the vasculature, were inoculated through intravenous injection to label the blood vessel and target cells before assembling the equipped mouse with a stereotactic instrument in the chamber (Fig. 1B).

In contrast, researcher can track a specific cell using a genetically modified mouse, which possesses indigenous fluorescence in a specific type of cell. It is now possible to understand the morphological and functional changes in immune cells during the immune response in the brain. For example, observations of actual immune phenomena under experimental neuroinflammatory conditions have been reported [19, 22, 23, 26]. However, due to a poor understanding of the blood-brain barrier (BBB), a brain-specific structure that prohibits most cells or substances from infiltrating the CNS, the brain is known to be an immune-privileged site [27–29]. However, recent studies have revealed various undisclosed facts regarding the brain via intravital imaging, including new discoveries related to the BBB and new hypothetical directions for further studies. For example, the permeability of the BBB has been measured using TPM under various conditions. In addition, it has been reported that the BBB can be flexible due to inflammation-related needs and stimuli [30]. Upon inflammation-related stimulation, leukocytes are recruited to the site via a cascade process. Briefly, during intravascular migration, leukocytes perform rolling, adhesion, and detachment in blood vessels. Following firm attachment to vascular endothelial cells, leukocytes begin extravasation via trans-endothelial migration [28, 31]. Some extravasated leukocytes can return to the blood vessel; this process is called reverse trans-endothelial migration [22]. The interaction of immune cells via signaling in inflammatory situations was also confirmed; in particular, neutrophil-microglia contact may play an essential role in neuroinflammation, which suggests the need to probe relevant molecular signaling pathways [26, 32]. Brain research through intravital imaging would provide opportunities to reveal unknown immunological phenomena and contribute to progress in preclinical research.

## Observation of Neutrophil Extracellular Trapin live mouse brain

**Fig. 2:**
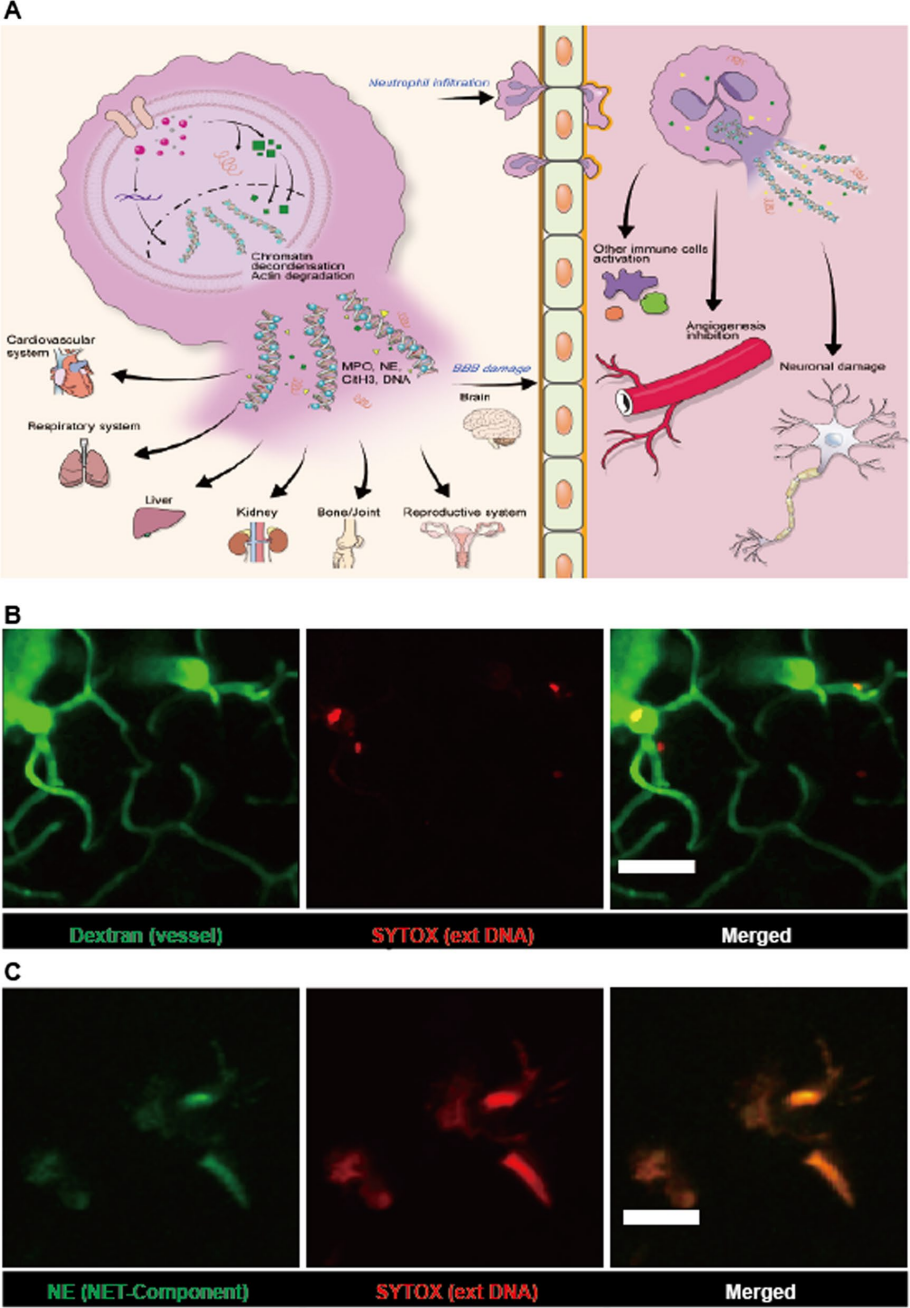
Formation of neutrophil extracellular traps (NETs). **A** Schema of NET formation. In the process of NET formation, various molecular components such as ROS, PAD4, NE, MPO, and citrullinated histone are involved. Once the membrane is ruptured, the intracellular component is emitted as tangled neutrophil components. This is called a NET. It can be found in any organ and location. Thus, NETs and their accompanied components are involved in neutrophil-gated immune response in multiple organs, including the brain. The image sets represent intravital imaging of NETs in the LPS-induced inflamed mouse brain conducted via two-photon microscopy. **B** The brain blood vessel was stained with FITC-dextran (green, 70 kDa, 2.5 mg/kg) and SYTOX-orange (red, 5 mM). As SYTOX labels DNA strands not covered with intact membranes, it is possibly used as a NET indicator (red). Scale bar: 50 μm. **C** NETs are visualized using two different NET-defining markers: SYTOX-orange (red, 5 mM) and neutrophil elastase–Alexa 488 conjugated antibody (green, 0.1 mg/kg). Neutrophil elastase is used as one of the components of the NETs. It is observed as tangled with extDNA stained with SYTOX. Scale bar: 20 μm

As a hallmark of inflammation, neutrophils are the first-line of leukocytes against pathogens in the early phases of invasion by rapid response [28]. They also perform highly versatile immune functions such as phagocytosis, migration, wound repair, inflammation mediation, communication with other immune cells, and extracellular trap generation [9, 33, 34]. Among these, neutrophil extracellular traps (NET) have recently become a topic of interest as a novel feature of neutrophils. NETs have been found to be an intrinsic defense function of neutrophils, capturing and holding pathogens [35]. ROS are generated in response to stimuli and activate other factors that cause NET generation such as MPO, neutrophil elastase, and PAD4. Following these reactions, chromatin is decondensed and histones are citrullinated. Finally, neutrophils release intracellular granules, which are the intracellular components containing neutrophil elastase, citrullinated histone complexes, and other granular proteins [35–38] (Fig. 2A).

However, recent studies have noted that NETs can function as a double-edged sword, inversely causing several diseases owing to their sticky properties and tendency to agglomerate, with several components of NETs possessing the potential to cause tissue damage [23, 39–42]. NET accumulation is implicated in systemic lupus erythematosus [11, 41, 43], thrombosis [44], rheumatoid arthritis [45, 46], and tumors [47], and is also pointed out as an etiological factor in CNS diseases, such as Alzheimer’s disease [48], stroke, and traumatic brain injury [49–51]. Although the accumulation of NET in several CNS diseases has been reported, their mechanism of aggravating and affecting diseases and inflammatory microenvironments remains largely elusive, especially in vivo [24, 49, 50, 52, 53]. NETs can be observed in real-time using two-photon intravital imaging to explore their effect in the inflammatory environment of the brain [11, 12, 23, 54–56]. There are well-known neutrophil stimuli that cause NET generation, such as phorbol 12-myristate 13-acetate, lipopolysaccharide (LPS), and ionomycin. Among these stimuli, we tested LPS and intravenously injected 2.5 mg/kg of it into wild-type C57BL/6J mouse. After 6 h, the brain was exposed, and intravital imaging was performed as described previously. Immediately before intravital imaging, SYTOX-orange (5 mM) was injected to visualize extracellular DNA (extDNA; the NET backbone) with FITC-dextran (70 kDa)–labeled blood vessels (Fig. 2B) [43, 56]. To accurately detect NETs, more than one specific marker such as citrullinated histones and neutrophil elastase can be used. In this study, we used SYTOX and neutrophil elastase–Alexa 488 conjugated antibody (0.1 mg/kg) for the accurate detection of NETs (Fig. 2C). This shows that NET imaging via two-photon intravital imaging is feasible and ideal for obtaining colocalized videos with three or four different NET-defining markers as well as blood vessels. NET visualization with different colocalized markers in intravital imaging is an ongoing area of study. Additionally, in most NET research articles, the structures of NETs in vitro have been described as bundles or spike-like. However, the majority of studies conducted in vivo showed NETs with a fluffy, round, or less sharp structure [23, 54–59]. This discrepancy in the shape of NETs between in vitro and in vivo conditions raise the possibility that the actual shape of NETs could be different from our knowledge. Thus, we could assume that NETs could have a different phenotype in vivo. Therefore, studying the controversial functions and shape of NET using intravital observation is crucial in understanding the real function and shape during the neutrophil-gated immune response, and applying TPM to NET research would be a cornerstone to delineate their undisclosed details.

## Conclusions

In this review, we summarized the general aspects of intravital brain imaging using two-photon microscopy and highlighted the advantages of two-photon intravital imaging for investigating the function and shape of NETs during the immune response by neutrophils in live mouse brains. Additionally, we discussed the possibility that NET generation in vivo could occur differently from that observed in vitro, and NETs may have disparate ways of interacting with neighboring cells and tissue components. Further studies using intravital observation of NET generation related to neutrophil function in inflamed brains would provide critical and more accurate information to investigate the actual mechanism of NET-mediated immune responses.

## Data Availability

For data and material, please contact ymhyun@yuhs.ac.

## Abbreviations

BBB: Brain blood barrier
NET: Neutrophil extracellular trap
SHG: Second-harmonic generation
TPM: Two-photon microscopy
